# Correspondence

**Published:** 1873-01

**Authors:** Thos. F. Rochester


### Correspondence.

### Utrum Horum Mavis?

PROF. MINER—*Dear Sir*:—In July, 1872, I was called to Le Roy to see Mr. Olmsted, of that village, in consultation with Dr. A. W. Fuller, his family physician. I found an intelligent gentleman, fifty-five years old, suffering from ascites and great general anasarca. He had been failing for about a year, his symptoms early indicating hepatic and gastric disorder. On the 3rd of February, 1862, the urine was first tested, and found to be highly albuminous, and this was afterwards always found to be the case, with the usual variation as to quantity. At the time of my visit dyspnœa, dizziness, and nausea, especially on rising in the morning, were the prominent symptoms. Debility, inappetence, and depression of spirits, also were marked. The heart sounds were feeble; but not abnormal. There was evident enlargement in the right hypochondriac region, with tenderness on percussion. Diagnosis: Granular kidney with hepatic complication; prognosis, unfavorable. Taking home with me a portion of urine, I made a careful microscopic examination, and found granular and fatty casts of the tubuli uriniferi in abundance, also free oil globules and granular matter. Diagnosis and prognosis confirmed.

Soon after my examination a physician (?) from New York, a foreigner, as I am informed, was sent for by Mr. O. Promise of cure was made. The visit was repeated three or four times, and enormous fees were paid. Mr. O. was tapped on the 20th of July, on the 4th of September, and on the 18th of October. Some sixty-three pounds of fluid were removed in the three operations. Death occurred a short time after the last paracentesis. On *post mortem* examination the heart was found to be healthy; the liver was enormously enlarged and cirrhotic. The kidneys were sent to me for examination. They were contracted, diminished in size; the capsule could not be separated from the gland, but brought away portions of it with it on removal. The malpighian tufts were ill-defined, and were impinged upon by the cortical structure. Portions scraped from the cut surface were examined microscopically, and beautiful specimens of clogged tubes were observed; also ruptured tubes, with their peculiar fungoid out-croppings; also oil globules and fat granules in large quantity.

Now, Mr. Editor, of what did the patient die? *Utrum Horum Mavis?* Unequivocally he had both renal and hepatic disease. The complication is so common that, to medical men, the question seems hardly necessary; but Dr. Fuller has been blamed by certain wiseacres in and out of the profession in his place of residence, they asserting that Mr. Olmsted did not have Bright's disease, "because he had a big liver."

These critics do not study, read or understand; they have eyes and see not. Allow me to refer them to two reasonably modern authorities, and many more if they desire: "In 250 cases of granular degeneration of the kidneys, the liver was cirrhosed in 37." (Dickinson on Albumenuria, 1868.) "Concomitant Complications—the most common is cirrhosis of the liver. It was present in 15 per cent. of my cases." (T. Grainger Stewart on Bright's Disease, 1869-1871.)

Trusting that this communication may not be without interest to your readers,

Yours respectfully, THOS. F. ROCHESTER.

